# Role of Molecular Targeted Therapeutic Drugs in Treatment of Breast Cancer: A Review Article

**DOI:** 10.1055/s-0043-57247

**Published:** 2023-05-23

**Authors:** Himanshu Singh

**Affiliations:** 1 Department of Oral and Maxillofacial Pathology and Oral Microbiology, Index Institute of Dental Sciences, Indore, Madhya Pradesh, India

**Keywords:** breast cancer, targeted therapy in breast cancer, therapeutic drugs in breast cancer, molecular targets in breast cancer, precision medicine in breast cancer

## Abstract

Breast cancer is a multifactor, multistage, and heterogeneous disease. Systemic treatment of breast cancer has changed significantly over the last decade. With a better knowledge of the pathogenesis, researchers and scientists have discovered numerous signaling pathways and synonymous therapeutic targets in breast cancer. Because of the molecular nature of breast cancer, which makes it difficult to understand, previous attempts to treat or prevent it have failed. However, recent decades have provided effective therapeutic targets for treatment. In this review, literature or information on various targeted therapy for breast cancer is discussed. English language articles were explored in numerous directory or databases like PubMed, Web of Sciences, Google Scholar, ScienceDirect, and Scopus. The important keywords used for searching databases are “Breast cancer,” “Targeted therapy in breast cancer,” “Therapeutic drugs in breast cancer,” and “Molecular targets in breast cancer.”

## Introduction

Breast cancer is a remarkably frequently diagnosed cancer in women globally. It poses a major risk to women's health and imposes a severe stress on individuals. There have been multiple progresses in breast cancer treatment in recent decades, but recurrence and progression occur after conventional treatments. The high rates are of serious concern and illustrate a requirement to develop new treatments for breast cancer. Recently, targeted molecular therapy is seen as a milestone in breast cancer precision medicine. 1 Malignant breast cancer is a compound molecular disease involving genetic modifications that control proliferation and growth of cells. The prevalent pattern of breast cancer is intermittently in character, with originally mutated oncogenes causing uncontrolled cell growth. 2 3

Although the advancement from normal to malignant breast tissue is not fully assumed, the mechanism is well assumed and therapeutics target the molecular modifications that appear during breast cancer development. Breast cancer is a heterogeneous illness with diverse pathological substance and diverse clinical management and molecular mutation that drive its growth, and feedback to therapy. 4 Chemotherapeutic agents feasible for the breast cancer treatment include plant alkaloids (vinorelbine, paclitaxel, and vindesine), alkylating agents (carboplatin, cisplatin, and cyclophosphamide), topoisomerase inhibitors (etoposide, irinotecan, and teniposide), and anthracyclines (epirubicin, doxorubicin, and idarubicin). Regardless of meaningful development in early discovery and substantial advances in treatment with systemic agents, breast cancers have acquired drug resistance. Currently, accepting the biological mechanisms and altered molecular events underlying carcinogenesis has directed to the recognition of new molecular targeted therapies. 4 In this review, literature or information on various targeted therapy for breast cancer is discussed. English language articles were explored in numerous directory or databases like PubMed, Web of Sciences, Google Scholar, ScienceDirect, and Scopus. The important keywords used for searching databases are “Breast cancer,” “Targeted therapy in breast cancer,” “Therapeutic drugs in breast cancer,” and “Molecular targets in breast cancer.”

## Targeting Epidermal Growth Factor Receptor Family

Epidermal growth factor receptor (EGFR) is a member of tyrosine kinases. EGFR participates in regulating proliferation of cell. EGFR family consists of four receptors named ErbB1/EGFR, ErbB2/HER2, ErbB3/HER3, and ErbB4/HER4. EGFRs are overexpressed in 40% cases of breast cancers and shows poor prognosis.

## EGFR Inhibitors

Cetuximab is a monoclonal antibody (Mab) that aggressively binds with domain of EGFR and restricts the receptor for activation of EGF ligand, which results in prohibition of intracellular signal transduction. Some preclinical studies suggested that cetuximab along with paclitaxel shows synergistic effect in typical case of breast cancer. 5 Various different in-progress clinical trials with cetuximab consist of association with various mediums like bortezomib, trastuzumab, irinotecan, and ixabepilone in triple-negative metastatic breast cancer (MBC).

Gefitinib reversibly suppresses EGFR autophosphorylation and constrains downstream signaling. Various clinical studies of single gefitinib and gefitinib plus hormonal therapy or chemotherapy for breast cancer are performed. The EGFR inhibitor gefitinib presented a low clinical benefit rate (CBR) and virtually no tumor response in patients with advanced breast cancer (ABC), regardless of whether their tumors were hormone receptor positive or hormone receptor negative. 6

Erlotinib is an additional EGFR tyrosine kinase inhibitor (TKI). In stage I study, when erlotinib was combined with trastuzumab, it resulted in anticancer activity and showed improved tolerance. When erlotinib was combined with docetaxel and capecitabine, it showed 67% of response rate in MBC patients. 7

## HER2 Inhibitors (Human Epithelial Receptor 2, also known as c-neu or c-erbB2)

Various different anticipating components are relevant with possibility of metastasis in breast cancer like as HER2, hormone receptor, and K _i_ -67 proliferation index. It is observed that HER2 overexpression is coupled with aggressive type and high mortality rate. 8 9

The principal recombinant bivalent humanized Mab is trastuzumab. It targets contrary to HER2 extracellular domain which results in blocking of intracellular signaling. Trastuzumab suppresses the tumor cells which overexpresses HER2. Trastuzumab is a humanized Mab which is used in treatment of breast cancer. It binds to HER2 and results in inhibition of downstream signal transduction. 10 The HERCULES study preliminarily assessed cyclophosphamide, Herceptin, and epirubicin in MBC and announced high tumor reaction with reduced cardiotoxicity. Clinical preliminaries have additionally affirmed that combining trastuzumab and anthracycline-taxane-based neoadjuvant chemotherapy in HER2-positive patients will bring about high rate of pathologic complete response. 11

In current researches, the blend of docetaxel, trastuzumab, and vinorelbine or capecitabine was highly dynamic as first-line combined treatment for HER2-positive metastatic breast carcinoma. The association of trastuzumab and capecitabine was dynamic and all around endured in patients with HER2-overexpressing malignant growth of breast impervious to both taxanes and anthracyclines and with overall survival (OR) rate of 22.3 and progression-free survival (PFS) of 4.1 months. 12 Then again, capecitabine plus trastuzumab and docetaxel as a first-line treatment was likewise found profoundly compelling with an OR of 25.5 months. 13

Pertuzumab is a Mab that ties to the extracellular HER2 dimerization area. It restrains the dimerization among HER2 and other HER relatives, particularly HER3, and furthermore enacts antibody-dependent cell cytotoxicity. 14 In spite of beginning viability, an enormous number of patients with MBC establish acquired resistance to trastuzumab. Besides, the incompetence of trastuzumab to traverse blood-mind boundary has been a significant explanation for metastasis of brain in patients on trastuzumab treatment. A reasonable choice to improve trastuzumab execution is to consolidate them with other transduction pathways inhibitors. Hence, little particle TKIs that mark HER2 overexpressing tumors and cross blood-cerebrum boundary could show action against metastasis of brain. Double EGFR-HER2 inhibitors for cancer of breast incorporate cetuximab, lapatinib, canertini, pertuzumab, and neratinib. 15

Lapatinib is an oral double TKI that inhibits HER2 and EGFR, by impeding the downstream pathways of signaling from these receptors. Lapatinib has been widely concentrated on in numerous clinical settings for treatment of patients with MBC. Studies have exhibited promising clinical action of lapatinib monotherapy in already untreated patients and those advanced on trastuzumab-containing treatment. Regardless of disease progression on earlier trastuzumab-based treatment, lapatinib in association with trastuzumab fundamentally further improved CBR and PFS versus lapatinib alone, in this manner offering chemotherapy-free option with a satisfactory well-being profile to patients with ErbB2-positive MBC. The most well-known treatment-related antagonistic occasions were diarrhea, rash, fatigue, and nausea. 15

Carey et al discovered that adding lapatinib to trastuzumab in addition to paclitaxel routine fundamentally improved the disease-free survival in breast cancer patients with HER2-positive. 16 Lapatinib monotherapy or in combination with capecitabine was found viable in patients with p95HER2-negative, HER2-positive, and p95HER2-positive cases of breast tumors. 17 In another review, lapatinib mixed with capecitabine was determined as a functioning treatment choice for women with HER2-positive MBC, incorporating those with moderate central nervous system (CNS) disease. 18

Afatinib, an anilino-quinazoline subordinate, is an oral double receptor TKI of EGFR and HER-2/neu. Afatinib was viewed as effective in contrast to cancers overexpressing EGFR with the secondary Thr790Met point change, which gives hindrance to and erlotinib and gefitinib. 19 Pertuzumab is a Mab that ties to an alternate epitope of HER2 than trastuzumab and intercept HER2 homodimerization and heterodimerization with other ErbB receptors. Single factor trials with pertuzumab were all around endured without notable unfavorable occurrence. In a stage II study of indiscriminate patients with HER2-negative illness pertuzumab monotherapy had restricted adequacy. 20

Neratinib is an orally controlled irreversible HER TKI. Initial researches have concluded antitumor movement of neratinib in patients recently treated with anthracyclines, trastuzumab, and taxanes. 21 Canertinib (CI-1033) is another unreversible pan-HER TKI that binds to the intracellular kinase domain's ATP-binding location covalently. Furthermore, it prohibits downstream signaling via PI-3 kinase/AKT pathways and Ras/MAP kinase. Canertinib, as an unreversible inhibitor, suppresses erbB receptor-mediated signaling for an extended period of time. 22

In recent times, tucatinib got endorsement in 2020 by the United States Food and Drug Administration for the treatment of HER2-positive MBC. Contrasted with different TKI, tucatinib is exceptionally particular, shown to be 1000-crease more intended for HER2. 23 24 Tucatinib is likewise found to have increases CNS infiltration than either neratinib or lapatinib, putting it at the front of conceivable HER2-positive MBC treatment. 24

## Targeting Vascular Endothelial Growth Factor Family

Angiogenesis assumes a significant part in development, attack, and metastasis in breast cancer. Vascular endothelial growth factor (VEGF) is a powerful inducer of migration of cell, intrusion, vascular permeability, and development of vessel. Five related glycoproteins, in particular, placental development factor, VEGFD, VEGFC, VEFGB, and VEGFA, act through three receptor tyrosine kinases VEGFR-3, VEGFR-2, and VEGFR-1. 25

## VEGF Inhibitors

Bevacizumab supervises in contrast to all isoforms of VEGFA. In a randomized trial, bevacizumab in addition to paclitaxel accomplished OR proportion of 48.9% versus 22.2% in paclitaxel-only group during first-line treatment of MBC. This study exhibited viability of bevacizumab in hindering angiogenesis. 26 In another comparable randomized trial, AVADO patients got docetaxel-only or in blend with bevacizumab as the first-line therapy of HER2-negative MBC. 27 Both the preliminaries showed critical upsurge in PFS ratio in patient getting bevacizumab. 27 In HER2-positive cases of breast cancer, consolidating trastuzumab with bevacizumab gives significant results. In an ongoing stage III preliminary, definitive chemotherapy was distinguished with chemotherapy added to bevacizumab, and chemotherapy with trastuzumab, carboplatin, and docetaxel with or without bevacizumab in breast cancer showing HER2 overexpression. These examinations were hopeful and gave experiences viewing utilization of bevacizumab as designated targeted treatment in HER2-positive cases of breast cancer. 28

## Multikinase Inhibitors

One more way to deal with hindering angiogenesis is to spot and suppress numerous individuals from receptor tyrosine kinases acknowledged to assume their part in proliferation of tumor and neovascularization. There are a few small molecules that go about as multikinase inhibitors. Sunitinib (SU11248) is a multi-TKI that targets PDGFR-α and -β, stem-cell factor receptor (c-Unit), VEGFR-3, VEGFR-2, VEGFR-1, and flt-3(fms-like tyrosine kinase receptor 3). These objectives assume significant part in the development and endurance of breast cancer. Preclinical examinations showed hindrance and relapse of breast cancers in animal models. In a stage II study, sunitinib monotherapy was well accepted and was tracked down to be very effective in patients with vigorously pretreated MBC. 29

Pazopanib, a multitargeted TKI, specifically hinders VEGF-mediated endothelial cell multiplication by focusing on VEGFR3, VEGFR2, and VEGFR1. Pazopanib displayed in vivo and in vitro movement in contrast to cancer development. In metastatic breast carcinoma, the mixture of lapatinib with pazopanib was more potent than lapatinib only. 30 Axitinib is an original TKI that targets all isoforms of VEGFR, c-KIT, and PDGFR. Axitinib was well acceptable in a stage I study with MBC patients. The regular side effects incorporate hypertension, fatigue, hand-foot syndrome, diarrhea, and proteinuria. Axitinib is used as first-line mixture because of its antiangiogenic activity and antitumor action with satisfactory safety profile. Stage II investigation of docetaxel with axitinib in MBC patients with no earlier chemotherapy had longer PFS contrasted with docetaxel in addition to placebo. 31 Vandetanib is a TKI that points EGFR and VEGFR2 to repress angiogenesis and growth of tumor. Vandetanib monotherapy was mostly well acceptable yet had restricted activity in patients with refractory MBC. 32

## Targeting RAS/MEK/ERK Pathway

The Ras superfamily is imperative regulatory switches engaged with cell expansion and distinction. Oncogenic Ras change happens under 5% yet overexpression of Ras protein has been depicted in breast cancer. Overexpression of Rho and Rac proteins has been related with increased intrusiveness and MBC. Ras proteins are accelerated by a few development factor receptors engaged in breast cancer like IGF-1, HER2, HER1, and ERα. Ras thus activates Raf/MEK/ERk and PI3K/Akt kinase flows that are engaged with cell endurance and expansion.

## Farnesyltransferase Inhibitors

The enzyme farnesyltransferase is convoluted in the posttranslational conversion of Ras and activates signal transduction. Therefore, it makes sense to use farnesyltransferase inhibitors (FTIs) to obstruct Ras conversion and downstream signaling as workable therapeutic object. FTIs are considered to have antitumor action beyond the Ras signaling pathway, as they further act on definite alternative proteins convoluted in cell regulation that desire farnesylation for their action. Tipifarnib is an oral FTI that obstructs farnesylation of Ras and alternative proteins convoluted in signaling pathways. Tipifarnib has demonstrated antitumor activity in in vitro and in vivo preclinical studies. A phase I single-agent trial was well acceptable with no indicative toxic effects. In a phase II trial, tipifarnib achieved a 10% partial feedback and definite cannabinoids in a patient with ABC. 33

## Raf Inhibitors

Raf is a subsequential effector of Ras and is phosphorylated to activate the MAPK (mitogen-activated protein kinase) cascade. Raf exists in three isoforms: Raf-1, B-Raf, and A-Raf. Mutant B-Raf has been identified in at least 7% of cancers. Sorafenib is a small-molecule blockage of Raf kinase action, along with alternative target molecules like flt3, VEGFR3, V3GFR2, VEGFR1, c-kit, and PDGFR-β thereby increasing cell proliferation, and simultaneously blocking neogenesis and apoptosis. A phase II investigation in MBC suggested that sorafenib resulted in steady disease and that sorafenib altered tumor growth progression rather than reducing tumor amount. Hence, the combination of sorafenib and standard of care is proposed with endpoints that are more conscious to the consequence of targeted agents. 34

## Targeting Cell Cycle and Apoptosis

### PI3K/Akt/mTOR Pathway Inhibitors

The PI3K/Akt passageway performs an essential part in various cellular activity along with proliferation, growth, survival, and angiogenesis. Uncontrolled stimulation of the present pathway by ER, IGF-1, and HER3 is behind breast cancer advancement and progression. Loss of oncogenic Ras and PTEN are other stimulators of the current pathway. It is also established that the Akt signaling pathway regulates the serine-threonine kinase and the mammalian target of rapamycin (mTOR).

### mTOR Inhibitors

Everolimus, an oral mTOR inhibitor formerly used to counter rejection after kidney or heart transplantation, has shown an encouraging understanding to conquered resistance to endocrine and targeted therapies in progressive breast cancer. Since mTOR is a downstream tumor signaling molecule that is associated with signaling pathways such as HER2 and hormone receptor, prohibiting mTOR activation affects upstream signaling that performs an important aspect in tumor cell proliferation. 35

Temsirolimus, a distinct mTOR inhibitor analogous to everolimus, was recognized in 2007 as an advanced treatment for renal cell carcinoma. 36 Temsirolimus additionally binds to FK506-binding proteins and the subsequent compound binds to mTOR and inhibits its effect on G1 phase progression of the cell cycle, cell growth, and proliferation. In particular, initial clinical trials are analyzing the effectiveness of temsirolimus as monotherapy in ER-positive, HER2-positive, or PTEN-deficient progressive breast cancer or MBC. 37 38

### PI3K Inhibitors

Available mTOR inhibitors obstruct cell expansion and proliferation, triggering AKT phosphorylation via feedback-activated pathways, which may lead to mTOR inhibitor resistance. However, considerable preclinical studies observed that PI3K inhibitors could hinder or eradicate AKT phosphorylation. Therefore, PI3K inhibitors may be clinically competent in individuals undergoing treatment with mTOR inhibitors. PI3K inhibitors principally comprised of α-specific PI3K inhibitors (taselisib and alpelisib) and pan-PI3K inhibitors (pictilisib and buparlisib). Buparlisib is a pan-PI3K inhibitor that aims all isoforms of type I PI3Ks. BELLE-322 was a stage III research to evaluate the effectiveness of buparlisib versus placebo in combination with fulvestrant after an aromatase inhibitor has progressed or has progressed to an mTOR inhibitor in postmenopausal women with HER2-negative and hormone receptor-positive progressive breast cancer. 39 Overall, recognized advanced treatment for postmenopausal women with HER2-negative and hormone receptor positive progressive breast cancer encompass the mTOR inhibitor everolimus added to tamoxifen or tamoxifen as first-line therapy, and palbociclib with fulvestrant or letrozole as second-line treatment. 40

Ipatasertib, an immensely particular oral small molecule AKT inhibitor, is being studied for effectiveness in contrast to tumors like triple-negative breast cancer. A phase 1 trial of ipatasertib in previously treated patients with multiple tumor types, including breast cancer, demonstrated an acceptable safety profile and preliminary antitumor activity. 41

Pictilisib (GDC-0941) is an orally accessible pan class I PI3K inhibitor currently being investigated in clinical researches for the treatment of progressive breast cancer. 42 43 Pictilisib nonspecifically inhibits all four isoforms of PI3K because it binds to the ATP (adenosine triphosphate)-binding pocket. It has also been displayed to be efficient against HER2 negative and positive cancers and PIK3CA mutant. Preclinical researches demonstrate increased “taxane antitumor activity” and increased apoptosis during treatment with pictilisib. 43 Pictilisib may also show anti-angiogenic effects because of growth inhibition observed when administered to activated human endothelial cells. Letrozole obstructs a principal molecule in the PI3K signaling pathway, an essential target for advanced drugs being matured to conquered resistance. In a stage II trial, letrozole plus temsirolimus alone or in consolidation prolonged PFS over letrozole-only in a patient with HER2-positive MBC. 44 An in vitro study showed that, NVP-BEZ235, a distinct PI3K/AKT/mTOR blocker, canertinib, and pacritinib, inhibited cancer cell growth in response to (irreversible) primary and secondary ErbB-targeted drugs. 45

## Cyclin-Dependent Kinase Inhibitors

Flavopiridol is a CDK (cyclin-dependent kinase) inhibitor and is seen in various researches as an antitumor agent. It is observed that flavopiridol sensitizes cells in breast cancer to TRAIL-induced apoptosis in vitro by promoting the prime recreation of the apoptotic pathway. A stage I study of docetaxel followed by flavopiridol showed promising clinical activity, even in individuals with densely pretreated taxanes. 46

### p53

Mutations in the p53 tumor suppressor gene are answerable for different human cancers, in addition to breast cancer. mtp53 (Mutant p53) protein converse counteraction to chemotherapeutic agents and promotes tumor cell endurance. Restoration of p53 activity is therefore an effective approach to advocate tumor cell apoptosis. PRIMA-1 is a safe minute molecule that modifies mtp53 to its active framework and activates tumor cell apoptosis. In in vitro research, therapy of mtp53-expressing breast cancer cells with PRIMA-1 resulted in dose-dependent mortality through a mitochondria-dependent apoptotic pathway. On the other hand, cells showing wtp53 protein were unaffected. 47

## Targeting Invasion and Metastasis

### Matrix Metalloproteinases Inhibitors

Matrix metalloproteinases (MMPs) are convoluted in infiltration of tumor and metastasis and are involved in the growth of breast, colon, ovarian, and lung cancers. In stage I trials, BAY 12–9566 (tanomastat), an inhibitor of MMP-9, MMP-3, and MMP-2, was well passable in individuals with solid tumors and shows no musculoskeletal consequences. 48

## Targeting src-Family Tyrosine Kinases

### src Inhibitors

The v-Src (Rous sarcoma virus) is a non-receptor allied with progression, development, and metastasis. Src is downstream of distinct growth agent receptors like IGF-1R, EGFR, HGFR, and PDGFR. Src activation is found in approximately 40% of ER-positive cancers and is acknowledged to be answerable for anti-estrogen resistance. Dasatinib is a tiny molecule inhibitor of Src and protein kinases consisting of c-Kit, PDGF-β, Bcr-Abl, and EphA2. It has been established to be competent in contrast to breast cancer cells. In vitro and early clinical studies demonstrated moderate efficacy as single agent in patients with triple-negative MBC. 49 Saracatinib (AZD-0530) is an eminently selective dual-specification small molecule inhibitor of Bcr-Abl and Src kinase. In a research, saracatinib in combination with fulvestrant was extra competent than either agent alone in inhibiting the growth of ER-positive breast cancer cells in vivo and in vitro. This research further showed that saracatinib monotherapy induced drug counteraction through ignoring the activation of mTOR pathway. Hence, using an mTOR kinase inhibitor together with saracatinib may be a feasible advantage to overcome drug counteraction and enhance effectiveness over saracatinib. 50

### HSP90 Inhibitors

Heat shock proteins (HSPs) are essential for cell survival beneath stressful conditions. HSP inhibitors are unusual agents that apply proapoptotic effects by inhibiting the binding of ATP to the ATP/ADP binding pocket of HSPs. Early HSP90 inhibitors, such as tanespimycin an geldanamycin, had drawback in terms of hepatotoxicity and solubility. In stage I trial, tanespimycin was well acceptable in consolidation with trastuzumab and was found to prohibit HSP90 activity in a patient with HER-2-positive breast cancer. 51

## Insulin-Like Growth Factor Inhibitors

IGF and IGF-IR play critical roles in survival and proliferation of cancer cell, conferring resistance to targeted and hormonal therapies in various tumor varieties, in addition to breast cancer. Therefore, a therapeutic treatment targeting IGF-IR could be a potent anticancer agent. IMC-A12, IgG1 Mab, attaches with IGF-IR with immense affinity and inhibits downstream signaling and ligand-dependent receptor activation. IMC-A12 further mediates deterioration and internalization of IGF-IR. In a study, IMC-A12 has been shown to restrain the growth of various tumors in addition to lung, pancreas, breast, and colon. 52

NDGA (nordihydroguaiaretic acid) is a phenolic aggregate that restrains HER2 and IGF-1R. In vitro studies advocated that NDGA induces PARP cleavage, deoxyribonucleic acid fragmentation, and caspase-3 cleavage. Combination therapy with trastuzumab and NDGA resulted in greater effectiveness in trastuzumab-refractory cells. Derivatives of NDGA are right away in clinical researches for various solid tumors. 53

## Histone Deacetylase Inhibitors

Histone deacetylase inhibitors (HDAC) are a new type of anticancer agents that prohibit tumor cell proliferation and bring about differentiation and/or apoptosis by interrupting with the activity of histone deacetylase. HDAC inhibitors are involved in the transcriptional downregulation and its responsive genes in ER-positive cancer cells. Valproic acid is well acceptable with epirubicin and has shown significant antitumor action in heavily pretreated individuals. 54 55

## CDK4/6 Inhibitors

Generally, approximately 75% of individuals with MBC usually receive endocrine treatment. Because resistance occurs in almost all individuals, there is an increasing focus on characterizing new advances to treat resistance to endocrine therapy. The CDK4/6/ and cyclin D complex is a key manager of cell cycle advancement and is closely associated with breast tumor development. Moreover, recent evidence demonstrate that CDK4/6 inhibitors enhance tumor immunogenicity through potential elimination of tumor cells, providing an explanation for new combination therapies involving CDK4/6 inhibitors and immunotherapy. 56

## Palbociclib

Palbociclib, which is particularly confined to the ATP binding location of CDK4/6 proteins, prevents Rb phosphorylation, inhibits E2F transcription factor release, and blocks the cell cycle between G1 and S phases, and ultimately cease the growth of tumor cells. The stage 3 palbociclib continuing trial in the handling of Breast Cancer (PALOMA)-238 study investigated the effectiveness of palbociclib along with letrozole against letrozole-only in postmenopausal female with HER2-negative and ER-positive progressive breast cancer. 57

## Abemaciclib

Abemaciclib is an oral, particular, short molecule interruption of CDK4/6 that crosses the blood–brain barrier and inhibits intracranial tumor cell proliferation. Abemaciclib monotherapy demonstrates clinical efficacy in patients with HER2-negative and refractory hormone receptor-positive progressive breast cancer. 58

## Conclusion

It is observed that molecular-targeted therapy has made great advancements in the treatment of breast cancer. Trastuzumab is considered the key element of targeted treatment for HER2-positive breast cancer, showing remarkable effectiveness in adjuvant and neoadjuvant treatment. Dual-targeted therapy with trastuzumab and pertuzumab represents a new step in targeted therapy. mTOR and CDK4/6 inhibitors have demonstrated competence to regress targeted drug resistance to some extent. As our understanding of breast cancer etiology improves, more efficient targeted drugs can be used to mitigate symptoms. With the number of new breast cancer drugs approved in recent years, it is understandable that there is quiet much to look progressively in the future of the treatment of breast cancer. The targeted therapies considered here have commutated the landscape of treatment of breast cancer and brought dreams to patients of breast cancer who are still searching for remedy of breast cancer.
